# The Multi‐Dementia Panel: A Tool to Biologically Define Alzheimer's Disease

**DOI:** 10.1002/alz70856_104833

**Published:** 2026-01-08

**Authors:** Yanaika S. Hok‐A‐Hin, Marta del Campo, David P. Salmon, Paula Desplats, Lisanne in ‘t Veld, Azzam Aladdin, Katharina Bolsewig, Douglas R. Galasko, Charlotte E. Teunissen

**Affiliations:** ^1^ Neurochemistry Laboratory, Department of Laboratory Medicine, Amsterdam Neuroscience, Vrije Universiteit Amsterdam, Amsterdam UMC, Amsterdam, Netherlands; ^2^ Hospital del Mar Research Institute (IMIM), Barcelona, Spain; ^3^ Barcelonaβeta Brain Research Center (BBRC), Pasqual Maragall Foundation, Barcelona, Spain; ^4^ Shiley‐Marcos Alzheimer's Disease Research Center, University of California, San Diego, CA, USA; ^5^ University of California, San Diego, La Jolla, CA, USA; ^6^ Department of Neurosciences, University of California, San Diego, La Jolla, CA, USA; ^7^ Center for Circadian Biology, University of California, San Diego, La Jolla, CA, USA; ^8^ Department of Pathology, University of California, San Diego, La Jolla, CA, USA; ^9^ Vrije Universiteit Amsterdam, Amsterdam, Netherlands

## Abstract

**Background:**

Alzheimer's disease (AD) shares clinical and neuropathological features with other forms of dementia, such as frontotemporal dementia (FTD) and dementia with Lewy bodies (DLB), making accurate diagnosis challenging. To improve diagnostic accuracy across diverse neurodegenerative dementias, we previously developed cerebrospinal fluid (CSF) biomarker panels specific to AD. These panels additionally monitor diverse biological processes (e.g., cellular remodeling, protein phosphorylation, exosome assembly, immune system, and vascular function). We aim to further validate our AD‐Diagnostic and AD‐Differential diagnostic biomarker panels in an independent international cohort to show their robustness and support clinical applicability.

**Method:**

The AD‐Diagnostic (ABL1, SDC4, MMP10, ITGB2, CLEC5A, THBD, and SPON2) and AD‐Differential diagnostic (ABL1, THOP1, ENO2, ITGB2, DDC, MMP7, and VEGRF3) panels are multiplex proximity extension assay (PEA) immunoassays, analyzed using the Olink Q100 instrument. Clinical validation was performed in patients from the Shiley‐Marcos Alzheimer's Disease Research Center and included controls (cognitively unimpaired; *n* = 109), MCI‐AD (*n* = 43), AD (*n* = 40), and a non‐AD group that included patients with FTD (*n* = 15) or Lewy body disorders (LBD; *n* = 22).

**Result:**

The AD‐Diagnostic panel showed increased protein concentrations in patients with AD compared to controls (ABL1, SDC4, MMP10, ITGB2, and SPON2) or the non‐AD group (ABL1, ITGB2). Some proteins were already increased at the MCI stage (SDC4, MMP10, ITGB2, SPON2). The AD‐Diagnostic panel discriminated AD from controls with high accuracy (AUC = 0.938, 95%CI: 0.885‐0.992; previous findings: AUCs = 0.96‐0.99). The AD‐Differential diagnostic panel showed higher protein concentrations in patients with AD compared to the non‐AD group (ABL1, THOP1, ENO2, ITGB2). An exception was DDC, which was highest in LBD. The AD‐Differential diagnostic panel discriminated AD from the non‐AD group with high accuracy (AUC = 0.845, 95%CI: 0.759‐0.931; previous findings: AUCs = 0.80‐0.87).

**Conclusion:**

The consistent results observed across multiple cohorts underscore the potential of these CSF biomarker panels to enhance differential diagnosis and detect biological changes of AD, potentially in the early stages of the disease. In addition to their diagnostic utility, these biomarker panels provide detailed insight into the underlying pathological mechanisms of AD, highlighting their potential as biomarkers for monitoring treatment responses in clinical trial settings.

